# A problem of self-isolation in Japan: The relationship between self-isolation and COVID-19 community case

**DOI:** 10.34172/hpp.2022.24

**Published:** 2022-08-20

**Authors:** Nam Xuan Ha, Truong Le-Van, Nguyen Hai Nam, Akshay Raut, Joseph Varney, Nguyen Tien Huy

**Affiliations:** ^1^Hue University of Medicine and Pharmacy, Hue University, Hue City, Vietnam; ^2^Online Research Club (http://www.onlineresearchclub.org), Nagasaki, Japan; ^3^Traditional Medicine Hospital of Ministry of Public Security, Hanoi, Vietnam; ^4^Division of Hepato-Biliary-Pancreatic Surgery and Transplantation, Department of Surgery, Graduate School of Medicine, Kyoto University, Kyoto, Japan; ^5^Global Clinical Scholars Research Training Program, Harvard Medical School, Boston, Massachusetts, USA; ^6^St. George’s Hospital, Grant Government Medical College and Sir J.J. Group of Hospitals, Mumbai 400001, India; ^7^American University of the Caribbean, School of Medicine, Sint Maarten, Sint Maarten; ^8^School of Tropical Diseases and Global Health, Nagasaki University, 1-12-4 Sakamoto, Nagasaki 852-8523, Japan

**Keywords:** COVID-19, Japan, Social isolation, Outpatients, Health policy

## Abstract

**Background:** The Japanese government advised mild or asymptomatic coronavirus disease-2019 (COVID-19) cases to self-isolate at home, while more severe individuals were treated at health posts. Poor compliance with self-isolation could be a potential reason for the new outbreak. Our study aimed to find out the correlation between the rising new cases of COVID-19 and home-based patients in Japan.

**Methods:** A secondary data analysis study was conducted with the data from COVID-19- involved databases collected from Johns Hopkins University, Japanese Ministry of Health, Labour and Welfare, and Community Mobility Reports of Google. New community cases, stringency index, number of tests, and active cases were analyzed. Using a linear regression model, an independent variable was utilized for a given date to predict the future number of community cases.

**Results:** Research results show that outpatient cases, the stringency, and Google Mobility Trend were all significantly associated with the number of COVID-19 community cases from the sixth day to the ninth day. The model predicting community cases on the eighth day (R2=0.8906) was the most appropriate showing outpatients, residential index, grocery and pharmacy index, retail and recreation index, and workplaces index were positively related (β_1_=24.2, 95% CI: 20.3– 26.3, P<0.0001; β_2_=277.7, 95% CI: 171.8–408.2, *P*<0.0001; β_3_=112.4, 95% CI: 79.8–158.3, *P*<0.0001; β_4_=73.1, 95% CI: 53- 04.4, *P*<0.0001; β_5_=57.2, 95% CI: 25.2–96.8, *P*=0.001, respectively). In contrast, inpatients, park index, and adjusted stringency index were negatively related to the number of community cases (β_6_=-2.8, 95% CI: -3.9 – -1.6, *P*<0.0001; β_7_=-33, 95% CI: -43.6 – -27, *P*<0.0001; β_8_=-14.4, 95% CI: -20.1– -12, *P*<0.0001, respectively).

**Conclusion:** Outpatient cases and indexes of Community Mobility Reports were associated with COVID-19 community cases.

## Introduction

 The global pandemic of coronavirus disease-2019 (COVID-19) began from an outbreak at China in 2019, it spread to worldwide later. During pandemic, multiple countries have been dealing with many new cases implicated as waves of infection. This is partly due to the early relaxation of control measures and restrictions. The appearance of variant strains of severe acute respiratory syndrome coronavirus 2 (SARS-CoV-2)^1,2^ have also crippled effectors to abolish the disease. Indeed, the delta variant has been reported as a dangerous factor given its high transmissibility and global spread. This variant has significantly increased the mean adequate reproduction number by 97 percent.^3^ Countries have employed a multitude of non-pharmaceutical interventions worldwide to ascertain control over the pandemic. Movement restrictions have been put in place in various degrees across 186 countries. Full or partial lockdown was constituted in 82 countries in the past year. In the absence of definitive treatment for COVID-19, many countries started developing vaccines against COVID-19. As of April 16th, 2021, there are 88 vaccines in clinical development and 184 vaccines in pre-clinical development. A few vaccines have already obtained emergency use authorization in multiple countries.^4,5^ However, the over-dependence on vaccines alone can be detrimental to areas stricken with poverty.

 A recent study suggests that over a fifth of the global population (low- and middle-income countries) would not access the existing vaccines until 2022.^5^ Hence, controlling the source of infection by breaking the chains of human-to-human transmission remains the best way to manage the outbreak. An essential method of controlling the source of infection is the immediate isolation of COVID-19 patients. In some countries such as Vietnam, the government has started to control the declaration of health status and history of the movement by using a BlueTooth-based mobile application that serves to caution its users with close contact with a COVID-19 patient.^6^ Either home-based or institution-based isolation accomplishes this. Institution-based isolation is better in controlling community transmission of COVID-19,^7,8^ though home-based isolation is understandably the preferred method. Another method is the early diagnosis of COVID-19 cases. The early diagnosis allows to immediately isolate and prevent the further spread of disease. However, it has been found that lack of timely diagnosis can significantly affect the primary reproduction number, increase the risk of transmission to the community leading to an outbreak of cases in the community.^9^

 Despite having an effective healthcare system alongside technological advances and a robust universal health insurance system for its citizens, Japan has been no exception to the pandemic. They are currently facing the fourth wave of COVID-19 infections^10,11^ and have declared a third state of emergency from April 25th to May 11th, 2021, ahead of the Olympic Game.^12,13^ This action appeared to express limitation on their strategy against COVID-19 regardless the availability of vaccine. Besides, the Japanese government has encouraged the citizens to avoid the “Three Cs” (3Cs) in daily life, including closed spaces with poor ventilation, crowded places with many people nearby, and close-contact settings.^14^ This policy is considered as one of the critical measures to prevent the occurrence of the new clusters. Along with 3Cs, the authority advised people having mild COVID-19 symptoms to self-isolate at home, and avoid work or public spaces.^15-17^ In contrast, more severe individuals were treated at health posts.^17^ Home isolation with mild cases was similar to nations at Europe and North America.^18,19^ However, the compliance to self-isolation measures is questionable as the findings of a recent study on Japanese workers showed that very few people practiced self-isolation.^20^ Therefore, it is likely that mild or asymptomatic cases could be an infectious source of new outbreak if they do not obey the government’s regulation. This study aimed to analyze the correlation between the raised community cases and home-based cases with mild or limited symptoms in Japan.

## Materials and Methods

###  Data sources

 This was a secondary data analysis study with data from three different databases during March 1st, 2020 to March 31st, 2021:

 COVID-19 database of Center for Systems Science and Engineering (CSSE) at Johns Hopkins University is an interactive web-based dashboard to visualize and track COVID-19 cases in real-time. It reports cases at province level in China, county level in the USA, state level in Australia, Canada, Japan, and at the country level otherwise. The location, number of confirmed cases, deaths, and recoveries was illustrated. The data source is collected from international organizations and local governments.^21,22^

- The COVID-19 dataset in Japan is an open nationwide data from Japanese Ministry of Health, Labour and Welfare. It includes demographic data based on the government website, daily information regarding the testing and medical care provision published by prefectures, and information regarding cluster events, obtained from the media or other sources. In this study, we analysed patient cases, number of tests and new community cases.^23^
- Community Mobility Reports is an anonymized Google database of movement trends over time by regions and place categories. It aims to gauge how people change in response to government policies during COVID-19.^24^ This data collected through Google Maps, and consisted of six different categories: residential, grocery and pharmacy, parks, retail and recreation, workplaces, transit stations in the form of changes comparing to the baseline period between January and February 2020. The daily data is affected by activities during the week, so we have converted the original data into the rolling seventh-day average data to evaluate the impact of policies.

###  Procedure 

 Indicators in the study are defined in Table 1.

**Table 1:** The definition of used indicators

**Indicators**	**Detail**
New community cases	New community cases are the number of new COVID-19 cases reported by the Japanese government.
Adjusted Stringency Index^25^	The stringency indexis an aggregate measure of nine metrics, including school closures, workplace closures, cancellation of public events, restrictions on public gatherings, closures of public transport, stay-at-home requirements, general information campaigns, restrictions on internal movements, and international travel controls to assess government responses. This index is calculated by the average scores of all nine indicators with values ​​between 0 and 100. Because government responses was affected by outbreaks, we used the adjusted stringency index to assess the government's response to the extent of outbreaks. $adjusted\;strigency\;index=\frac{Stringency\;index\;(dayi)}{Active\;cases\;(1000\;cases)(dayi)}$
Adjusted Number of Tests	Because the number of tests was affected by the outbreak, we used adjusted tests. Our research assumed that "Adjusted number of tests" could help detect infections at present, but detected and controlled cases would probably reduce the infection in the future. $Adjusted\;number\;of\;tests=\frac{Test\;(dayi)}{Active\;cases\;(1000\;cases)(dayi)}$
Actual Active Cases	Because the SARS-CoV-2 virus can transmit directly human-to-human, the source management is crucial to control outbreaks. In our study, the source of infections as actual active cases has been identified, including active cases (Active cases = confirmed cases - death cases - recovered cases) and cases that have been tested but have not been reported (this time in Japan is two days). Furthermore, these cases have been classified into two groups according to specific patient management status as follows: Inpatient cases are the number of COVID-19 infections required for hospital isolation and treatment as reported by the Japanese government. Outpatient cases are confirmed COVID-19 patients with mild or asymptomatic presentation who are asked to self-isolation at home. In this study, outpatients was defined as: Outpatient cases = Actual active cases – inpatient cases Notably, this indicator was affected by several important components such as under reporting of cases, PCR test accuracy, and the detection rate that was extremely low for mild cases.
Community Mobility Reports	It included six categories: residential, grocery and pharmacy, parks, retail and recreation, workplaces, transit stations.

###  Statistical analysis

 As of March 31st, 2021, the proportion of the population vaccinated in Japan has been at 0.69%, and SARS-CoV-2 is human-to-human, so we assumed that the community cases would be directly affected by Actual Active Cases, Government’s policy (Adjusted Stringency index), people’s compliance based on Community Mobility Reports, and Adjusted Number of Tests.^26^ Although we all knew that each infected case was in interaction, for this study, we also assumed that the data was independent and only affected by Actual Active Cases, Stringency Index, Number of Tests, and Community Mobility Reports. Therefore, we utilized an independent variable for a given date to predict the future number of community cases using a linear regression model. Based on the incubation period of SARS-CoV-2, a mean of 5.2 days and a 95% confidence interval of 4.1 to 7.0 days, plus a late reporting time of 2 days (corresponding to test result waiting time and update data delay in Japan).^27^ Our study assumed that the number of cases (inpatient cases, outpatient cases), the adjusted number of tests, the adjusted the stringency index, and Google Mobility Trend (Residential index; Grocery and pharmacy index; Retail and recreation index; Parks index; Workplaces index; Transit stations index) were related to the number of new community cases at 6^th^, 7^th^, 8^th^, and 9^th^ day later.

 We used Bayesian model averaging - an application of Bayesian inference to determine the optimal model based on R^2^ value, BIC index, and Post-Prob index to select variables in the aforementioned linear model. The analysis was implemented on Bayesian Model Averaging (BMA) package by R software version 4.0.3 (CRAN main site at https://CRAN.R-project.org).

## Results

**Figure 1:**
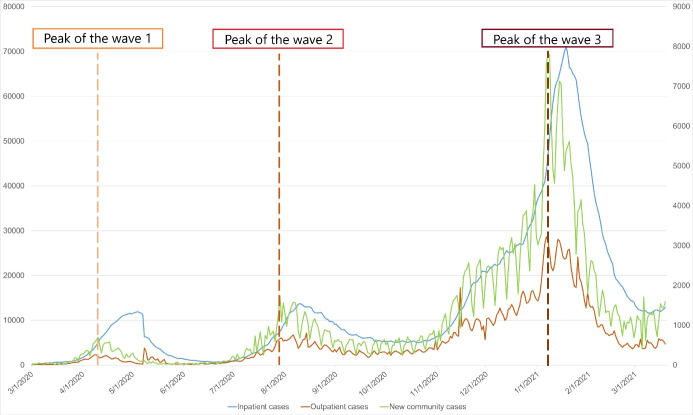
Time series of the number of new community cases, inpatient cases, and outpatient cases. New community cases, inpatient cases, and outpatient cases were represented in light blue line (secondary y-axis), blue line, and red line (primary y-axis), respectively. The peak of COVID-19 outbreaks with the highest community cases corresponded with the top of outpatient cases

Figure 1 shows the time series of the number of new community cases, inpatient cases, and outpatient cases. The peak of COVID-19 waves with the highest community cases corresponded with the top of outpatient cases. While outpatients had stationary, the community cases decreased rapidly. This chart also presents that the gap between inpatients and outpatients widened during outbreaks and narrowed during stable community cases.

**Figure 2:**
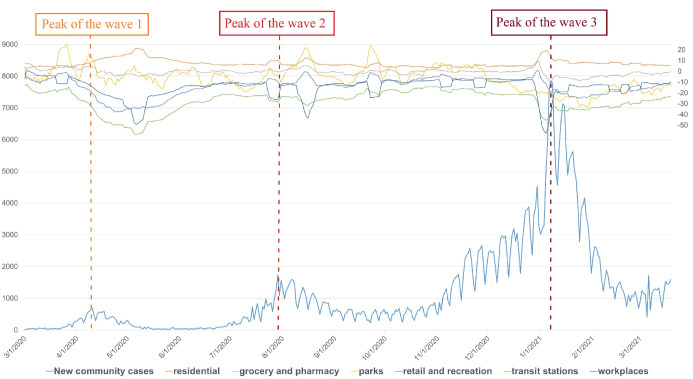
The time series of Community Mobility Reports and the number of new community cases. The blue line represents the new community cases (primary y-axis), while the secondary y-axis depicts six indicators of Community Mobility Reports with different colors

 The timeline of an association of Community Mobility Reports and new community cases was described in Figure 2. Eight days before the peaks of waves, retail and recreation index, transit stations, workplaces started to decline, and the lowest levels are strikingly similar to the second and third waves. Meanwhile, the grocery and pharmacy index and parks index increased in the first and second waves, but decreased slightly in the third wave. In contrast, the residential index tended to grow over time and rapidly increased during waves.

**Figure 3:**
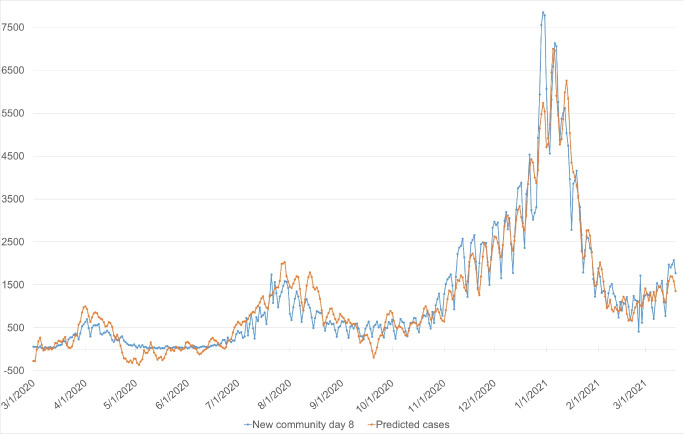
Predictive values ​​of the model on the eighth day and the number of community cases in real time. The predicted value was relatively same as the actual value

Table 2 shows the predictive model community cases corresponding to the incubation periods from 4 to 7 days. The model predicted the number of community cases on 8^th^ day (matching the incubation period of 6 days and 2 days waiting to report results) was the best-fitting model. It achieved the highest value of R2 (R2 = 0.89). Figure 3 emphasized the predictive values ​​of this model in the forecast of community instances. In this model, an increase of 100 outpatient cases related to the apparition of approximately 24 community cases. Furthermore, it was revealed that the increment of 1% of Community Mobility Reports index in residential, grocery and pharmacy, retail and recreation, the workplace would escalate the new community cases by 278, 112, 73, and 57 cases, respectively. For the parks index, the elevation of one unit of adjusted the stringency index, the increase of every 100 inpatient cases, and the increment of 1% of Community Mobility Reports would significantly reduced community cases (14, 3, and 33 points, respectively). Figure 3 with the number of new community cases on the eighth day and the forecast from the best-fitting model showed that the predicted value was relatively same as the actual value, even when the number of new community cases broke out and the periods of this indicator was stable.

**Table 2:** The predictive models of community cases corresponding to an incubation period of 4 to 7 days using BMA package^a^

**Periods of prediction (incubation+2 days of reporting)**	**6** ^th^ ** day (4+2 days)**			**7** ^th^ ** day (5+2 days)**			**8** ^th^ ** day (6+2 days)**			**9** ^th^ ** day (7+2 days)**		
	**Beta**	**95% CI**	***P***	**Beta**	**95% CI**	***P***	**Beta**	**95% CI**	***P***	**Beta**	**95% CI**	***P***
Intercept	298.2	-44.7; 641.1	0.088	92.9	-63; 248.8	0.242	119.3	46.9; 378.6	0.126	212.7	46.9; 378.6	0.012
Adjusted the stringency index	-13.4	-17.4; -9.3	< 0.0001	-13.3	-17.1; -9.4	< 0.0001	-14.4	-20.1; -12	< 0.0001	-16.1	-20.1; -12	< 0.0001
Adjusted the number of tests^b^	47.8	0.9; 94.6	0.046	-	-	-	-	-	-	-	-	-
Outpatient cases (100 cases)	18.6	17.3; 20	< 0.0001	22.2	19.4; 24	< 0.0001	24.2	20.3; 26.3	< 0.0001	23.3	20.3; 26.3	< 0.0001
Inpatient cases (100 cases)	-	-	-	-1.80	-2.9; -0.7	0.001	-2.8	-3.9; -1.6	< 0.0001	-2.7	-3.9; -1.6	< 0.0001
Residential	385.8	265.7; 505.9	< 0.0001	288.7	177.5; 399.8	< 0.0001	277.7	171.8; 408.2	< 0.0001	290	171.8; 408.2	< 0.0001
Grocery and pharmacy	94.3	50.4; 138.2	< 0.0001	96.2	59.3; 133.1	< 0.0001	112.4	79.8; 158.3	< 0.0001	119	79.8; 158.3	< 0.0001
Retail and recreation	58	20.3; 95.6	0.003	77	52.8; 101.3	< 0.0001	73.1	53; 104.4	< 0.0001	78.7	53; 104.4	< 0.0001
Parks	-28.4	-37.3; -19.5	< 0.0001	-30	-37.8; -22.2	< 0.0001	-33	-43.6; -27	< 0.0001	-35.3	-43.6; -27	< 0.0001
Workplaces	61.9	25.7; 98.2	0.001	59.2	25.6; 92.8	0.001	57.2	25.2; 96.8	0.001	61	25.2; 96.8	0.001
Transit stations	46.4	5.5; 87.2	0.03	-	-	-	-	-	-		-	-
**R2**	0.8743	0.8864	0.8906	0.8711

Abbreviations: BMA, Bayesian Model Averaging; CI, confidence interval. ^a^ In the model, 10 variables were analysed at the exposure date (day 0) to predict the number of cases diagnosed after the mean incubation period (from 4^th^ to 7^th^ day). For example, on the sixth day, the model forecasted the COVID-19 cases with incubation of 4 days and reporting of 2 days. The optimal model was determined by R2 value, BIC index, and Post-Prob index to select influence variables in the aforementioned linear model. *b*The proposed model on the 7^th^, 8^th^, and 9^th^ day didn’t comprise the “Adjusted number of tests” variable because this index wasn’t statistically significant and reduced the quality of the model.

## Discussion

 We focused on analyzing the number of COVID-19 outpatients having mild or moderate symptoms who were self-isolated and treated at home. Our result indicated that the outpatients could become a major factor causing the new outbreaks in Japan. The highest outpatient cases were respective to top of COVID-19 waves from a predictive model of community cases in the past outbreaks (an incubation period of 4 to 7 days^27-29^). The model showed that outpatients contributed significantly to the number of community cases. Among the predictive models, the analysis on the 8^th^ day was the most appropriate when it showed a strong association between government reports and an incubation period of 5.2 days plus 2 days to wait the announcement. Proposed models on the 7^th^, 8^th^, and 9^th^ day did not comprise the Adjusted Number of Tests variable because this variable was not statistically significant, even reduced the quality of the model.

 Our hypothesis found that a tremendous gap has been happening in the self-isolation of Japan since the COVID-19 pandemic emerged. The Japanese government could overlook the potential role of outpatients as an important infection source. The regulation only requires hospitalization for severe cases or high-risk patients. Other cases could be managed at home or accommodation facility.^17^ Although the public health centers took responsibility for outpatients, it might be ineffective because the rule of isolation could be broken anytime. The reason could be several factors. We inclined that the poor attitude and low knowledge were key elements. The government needed to consider a better strategy of health communication to promote knowledge and attitude of COVID-19 prevention together with responsibility. On the other hand, supervising outpatients with strict measurements was another strategy, for example, quarantining COVID-19 mild cases or outpatients in isolation facilities like Vietnam or control through follow-up application in South Korea.^30-32^

 In terms of movement, it was necessary to manage residential and retail strictly and recreation activities; however, opening the parks could be considered as long as ensuring the social distance. Studies showed that SARS-CoV-2 was at higher risk of infection in closed spaces, high population densities where exposure distances greater than 2 meters can hardly be guaranteed. During social distancing due to COVID-19, activities would be increased at residential areas or parks. In a residential area, activities among neighbours can make it challenging to maintain the six-feet distance, but it can be easier reached if activities are in the park. Therefore, visiting parks may be a good alternative for those seeking entertainment.^33,34^

 We also offered that the government measure policies against outbreaks by designing or applying a specific index like the Stringency index. In our study showed that Government Stringency Index should be adjusted according to the number of active cases to avoid the possibility of too tight or too loose application that negatively affects social performance and epidemic control.

###  Propose solutions

 Japan employed a variety of strategies in an attempt to control over the past COVID-19 waves. However, the potential disadvantage in their strategy could pose new outbreaks. One of them was effective management of COVID-19 outpatients. Here, we suggest several supplement measurements to prevent community transmission. Our solutions are not absolutely appropriate but they deserve to become valuable references for the governments. We hope that they will help Japanese avoiding a state of emergency in the future.

 First of all, improving knowledge, attitude of COVID-19 prevention and better self-isolation management.

**Figure 4:**
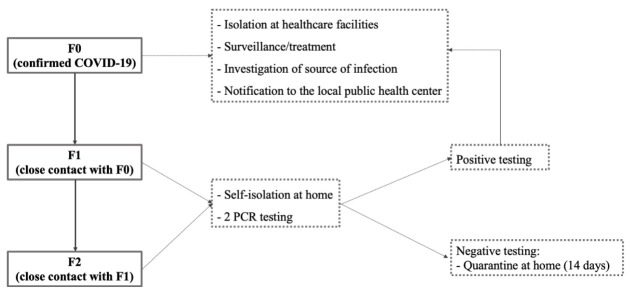
Proposal of COVID-19 tracing strategy for Japan

**Figure 5:**
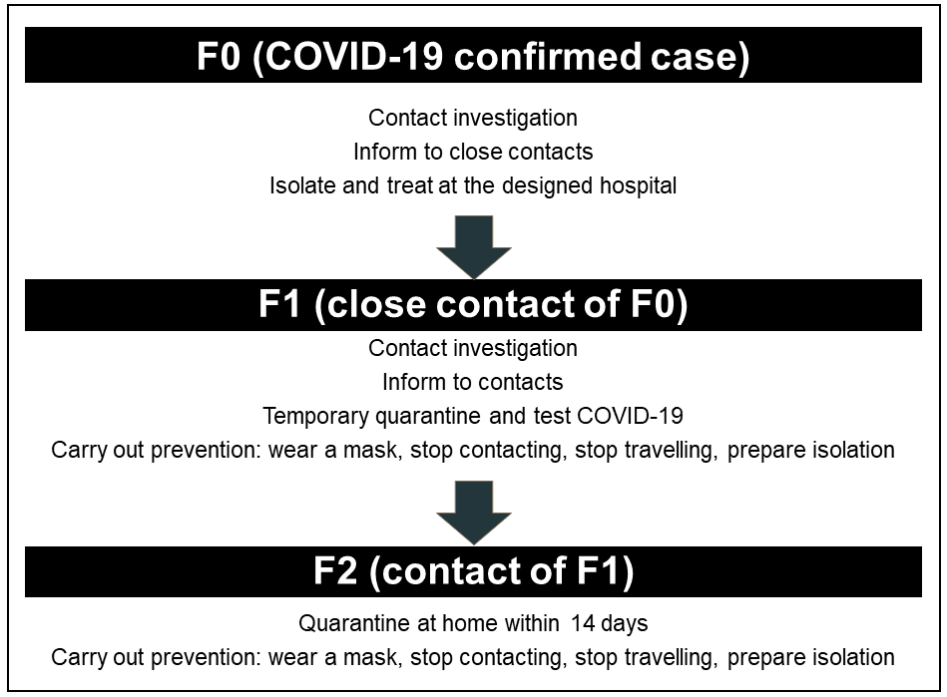
Subjects of COVID-19 tracing strategy

 Secondly, the intensive tracing could be a critical solution to pass the new outbreaks without an emergency state although this is not induced by our results. In more details, the tracing should not only focus on closed contacts but also expand their contacts as well.^6,35^ The closed contacts should test via PCR and stay home until the result is available (Figure 4 and 5). The undeniable efficacy of the Japan cluster-based approach will surge, paying more attention to F2 (contact of closed contact with confirmed cases), who were ignored. This might pose the silent transmission in case the tracing was not performed. The efficacy of F2 monitoring appeared in Vietnam, where the classification procedure of COVID-19 defines patients and their contacts from F0 to F5 (first generation of infection, second to fifth generation of infection).^6,35^ During the period of March to April 2020, a successful containment of a localized outbreak of COVID-19 in Bach Mai Hospital, Vietnam involved identification of 27893 F1 and F2 contacts who were traced, tested and quarantined at centralised centres; and about 24346 F3 and F4 cases were traced and quarantined at home.^36^ As of April 18th, 2021 with 2784 COVID-19 positive cases, the Government of Vietnam has already quarantined more than 15.7 million people.^37^

 Finally, early detection can be achieved by modifying the current medical declaration used by Japan called COCOA (Contact-Confirming Application) which is a smartphone application employing the principle of bluetooth and needs user approval to receive notification about potential contact within 1 meter for more than 15 minutes with a person who tested positive for COVID-19 whilst maintaining anonymity.^38^ Now, it has been applied to positive polymerase chain reaction (PCR) cases. We thought the application should cover all individuals with fever or flu-like syndrome (cough, shortness of breath, and runny nose). Besides, we can apply social network analysis (SNA) for analysing reported locations from like-flu symptomatic individuals to detect suspicious transmission. We can use SNA software packages (UCINET or Gephi) to explore the centrality of connections, and location detection can be simply completed.^39^ As a result, the early detection of COVID-19 clusters, as well as influenza, could be achieved.

## Conclusion

 Outpatient cases and Community Mobility Reports including residential index, grocery and pharmacy index, retail and recreation index, workplaces index were positively associated with COVID-19 community cases. It implied that isolation policy and compliance were closely related to the increase of community cases. It recommended that governments should manage effectively outpatients with mild or moderate symptoms and asymptomatic case with positive test, and evaluate the compliance through the Google Mobility Trend index to make appropriate adjustments.

## Acknowledgments

 We would like to appreciate all the teams of Center for Systems Science and Engineering (CSSE) (Johns Hopkins University), Japanese Ministry of Health, Labour and Welfare, and Google for public data of COVID-19. We also thank Editors and Reviewers of the Journal Health Promotion Perspectivesfor their valuable comments.

 Nam Xuan Ha^1,2#^, Truong Le-Van^2,3#^, Nguyen Hai Nam^2,4,5^, Akshay Raut^2,6^, Joseph Varney^2,7^, Nguyen Tien Huy^2,8*^

## Authors’ contributions

 NXH, TLV, and NTH made substantially contributions to the study’s conception and design. TLV analysed the data, while NXH and TLV interpreted the results. NXH reviewed the manuscript after all authors participated in the writing. NTH critically revised all works. All authors read and approved the final manuscript.

## Funding

 No funding was available in this study.

## Ethical approval

 Not applicable.

## Competing interests

 All authors declare that no competing interests exist
